# Prevalence of frailty among community-dwelling elderly persons in Spain and factors associated with it

**DOI:** 10.1080/13814788.2019.1635113

**Published:** 2019-10-22

**Authors:** Francisco Rivas-Ruiz, Mónica Machón, Eugenio Contreras-Fernández, Kalliopi Vrotsou, María Padilla-Ruiz, Ana Isabel Díez Ruiz, Yolanda de Mesa Berenguer, Itziar Vergara, Group GIFEA

**Affiliations:** a Unidad de Investigación, Agencia Sanitaria Costa del Sol, Marbella, Spain;; b Red de Investigación en Servicios de Salud en Enfermedades Crónicas (REDISSEC), Madrid, Spain;; c Unidad de Investigación APOSIs Gipuzkoa, Osakidetza, Donostia-San Sebastian, Spain;; d Instituto Biodonostia, Donostia-San Sebastian, Spain;; e Área de Investigación – Unidad de Gestión Clínica Prevención, Promoción y Vigilancia de la Salud. Distrito Sanitario Costa del Sol. Servicio Andaluz de Salud, Málaga, Spain;; f Centro de Salud Beraun, OSI Donostialdea, Osakidetza, Renteria, Spain;; g Unidad de Gestión Clínica de la Lobilla, Distrito Sanitario Costa del Sol, Servicio Andaluz de Salud, Estepona, Málaga, Spain

**Keywords:** Frailty, older adults, cross-sectional, prevalence, primary healthcare

## Abstract

**Background:** For effective prevention and intervention, and reduction of dependency, it is essential to determine the presence of frailty in the community.

**Objectives:** To describe the prevalence of frailty among elderly persons living independently, in two primary healthcare areas in Spain; to identify factors correlated with its presence.

**Methods:** This descriptive cross-sectional study was conducted between May 2015 and July 2016 among non-institutionalized individuals aged ≥70 years living in the primary healthcare areas of Gipuzkoa and Costa del Sol (Spain). The main outcome variable was the prevalence of frailty (determined by modified Fried criteria). The independent study variables were sociodemographic characteristics, anthropometric data and health-related life habits.

**Results:** The study population consisted of 855 individuals (53% women). The overall prevalence of frailty was 26.2% (Gipuzkoa 14.2%, Costa del Sol 38.0%). Using multiple logistic regression, the following factors were associated with frailty: female sex (OR: 1.98; 95%CI: 1.37–2.86); cumulative illness rating scale (OR: 1.05; 95%CI: 1.00–1.10); self-perceived health status (OR: 0.96; 95%CI: 0.95–0.97); self-perceived unhealthy lifestyle (OR: 3.37; 95%CI: 2.05–8.87); dissatisfaction with the domestic environment (OR: 2.11; 95%CI: 1.18–3.76); and cognitive impairment (OR: 4.10; 95%CI: 2.05–8.19). In the multivariable model, ‘geographical area’ differences persisted, with an OR of 3.51 (95%CI: 2.29–5.36) for the Costa del Sol area, using Gipuzkoa as reference.

**Conclusion:** In this population of community-dwelling persons aged 70 years and over, the prevalence of frailty was 26%. Factors correlated with frailty were female sex, comorbidity, poorer self-perceived lifestyle and health status, and dissatisfaction with the domestic environment.

KEY MESSAGES

- In this sample of men and women, aged ≥70 years and living independently in the community, the prevalence of frailty was 26%
- There were significant differences between both studied geographical areas
- Women presented frailty twice as often as men

## Introduction

Among the physiological changes of aging, frailty is a multidimensional clinical condition characterized by a progressive decrease in reserve capacity and adaptability, which is reflected in a global deterioration of health when exposed to a stressor [1]. The vulnerability of a frail elderly person is a dynamic condition that heightens the risk of adverse events such as falls, hospitalization, inability to perform basic life activities and premature mortality [2]. Among definitions of frailty is the phenotype proposed by Fried et al., widely used in research, based on the presence of low gait speed, reduced grip strength, decreased activity level, fatigue and unintentional weight loss [3].

The prevalence of frailty in the community varies greatly. In recent years, values ranging from 4 to 59% have been reported, both in developed countries (such as Spain) and in less developed ones [4–7]. In all countries, overall levels of frailty are higher among women, and there is an inverse relationship between frailty and education, and between frailty and economic level. However, differences have been observed according to the health system available and the social support received.

Community identification of frailty is essential to ensure effective preventive interventions for age-related conditions, and consequently to prevent dependency [8]. This question is granted high priority in health authority agendas [9].

This study compares the prevalence of frailty in two primary healthcare (PHC) areas (the organizational structure for the administration and provision of PHC in Spain), located within the same country (Spain), but in different geographical areas (north vs south).

The main purpose of this study is to describe the prevalence of frailty among elderly persons living independently, in two PHC areas in Spain, and then to identify sociodemographic characteristics, anthropometric data and health-related life habits.

## Methods

### Study design

This descriptive cross-sectional study was conducted from May 2015 to July 2016, addressing a multicentre prospective cohort with two years of follow-up, according to a previously described method [10]. The study was conducted in two PHC areas in Gipuzkoa and western Costa del Sol (northern and southern Spain, respectively). The characteristics of these PHC areas are summarized in Table 1. The research unit located in each area designed, coordinated and implemented the study.

**Table 1.** Descriptive analysis of the study population.

	PHC	
	Gipuzkoa	Costa del Sol
Reference Population	385 000	474 000
Elderly people (%)	11.9	11.1
Urban area^a^ (%)	44	100

^a^Recruited population resident in towns with >50 000 inhabitants.

### Setting and study population

The following inclusion criteria were applied: all participants should live within the community, be functionally independent (Barthel’s Index > 90 points), be aged 70 years or more and provide signed informed consent (Supplementary Appendix A). Persons who were terminally ill, or who spent less than six months per year in their residence or who had difficulty in communicating fluently in Spanish were excluded.

Potential participants were selected randomly from the administrative databases of the participating PHC (using the census of individuals registered to receive PHC, not the general population census) according to criteria of representativeness by age and sex. All subjects thus selected were contacted by letter and by phone and given information on the project. Those who expressed interest in taking part took the Barthel test by phone, and those whose test result was less than or equal to 90 were excluded.

In each PHC area, a trained nurse, the same person in every case, made the baseline assessment of the study subjects, via a face-to-face interview in which the dependent and independent variables were assessed.

### Variables

*Dependent variable: frailty*. The main outcome variable was the presence of frailty, assessed according to a modified version of the following criteria proposed by Fried:

1. Unintentional weight loss (over 4.5 kg) in the last year.
2. Low level of physical activity, identified if the person presented one of the following conditions: (a) walk less than 30 min continuously every day, three times a week; (b) do less than 20 min of sports activity, once a week; (c) have difficulty carrying the daily shopping.
3. Fatigue or lack of energy, measured by an affirmative response to either of these items on the CES-D Scale: ‘I felt that everything I did was an effort;’ ‘I could not get going’ [11].
4. Muscle weakness, identified according to the test, ‘Get up from a chair and sit down again, five times, as fast as possible’ [12]. If this task took 12 s or more, the criterion was considered to be met.
5. Slowness in mobility, measured using the timed get up and go test [13]. If this task took 10 s or more, the criterion was considered to be met.

Participants were identified as frail if they presented three or more of the above criteria, and were otherwise robust.

*Independent variables.* The independent study variables were sociodemographic characteristics, anthropometric data and health-related lifestyle. Table 2 categorizes the study variables considered. Self-perceived health status was evaluated with the EQ-5D-3L visual analogue scale (VAS; 0 (worst) – 100 (best)). Cognitive status was assessed using the mini-mental status examination (MMSE) scale (0–30 points; normal: 27–30; slight deficit: 23–26; cognitive impairment: <23). Comorbidity was assessed using the cumulative illness rating scale, CIRS; 0 (absence)–56 points (worse comorbidity status). Finally, the number of daily medicinal drugs was recorded.

**Table 2.** Descriptive analysis of the study population, total and segmented by geographical area.

	Total *n* = 855 *n* (%)	Gipuzkoa *n* = 423 *n* (%)	Costa del Sol *n* = 432 *n* (%)	*P*
Sex				
Male	402 (47.0)	184 (43.5)	218 (50.5)	0.049
Female	453 (53.0)	239 (56.5)	214 (49.5)	
Age				
Mean/SD	78.1 (5.0)	80.1 (4.2)	76.2 (4.9)	<0.001
Education background^a^				
None or only primary	680 (80.9)	338 (82.6)	342 (79.2)	0.233
Secondary or university	161 (19.1)	71 (17.4)	90 (20.8)	
Marital status^b^				
Married—civil partnership	541 (64.1)	250 (60.7)	291 (67.4)	0.024
Single	38 (4.5)	15 (3.6)	23 (5.3)	
Separated—divorced—widowed	265 (31.4)	147 (35.7)	118 (27.3)	
Family income^c^				
≤€1200	501 (61.5)	246 (64.1)	255 (59.3)	0.186
> €1200	313 (38.5)	138 (35.9)	175 (40.7)	
BMI^d^				
Mean/SD	28.9 (4.3)	28.2 (4.1)	29.6 (4.4)	<0.001
Smoking^e^				
Non-smoker	478 (56.0)	253 (60.0)	225 (52.1)	0.037
Ex-smoker	322 (37.7)	141 (33.4)	181 (41.9)	
Smoker	54 (6.3)	28 (6.6)	26 (6.0)	
Cumulative illness rating scale (0–56)^f^				
Mean/SD	6.6 (3.7)	5.1 (2.9)	8.1 (3.8)	<0.001
Number of medicinal drugs taken^g^				
Mean/SD	5.4 (3.3)	5.1 (3.4)	5.8 (3.2)	0.002
Self-perceived lifestyle^h^				
Healthy	727 (86.1)	368 (89.1)	359 (83.3)	0.019
‘Intermediate’—unhealthy	117 (13.9)	45 (10.9)	72 (16.7)	
Health status (0–100)				
Mean/SD	75.0 (17.2)	73.9 (15.2)	76.1 (19.0)	0.069
Satisfaction with the domestic environment^i^	** **	** **	** **	
Satisfied	768 (90.9)	386 (93.2)	382 (88.6)	0.027
Unsatisfied	77 (9.1)	28 (6.8)	49 (11.4)	
Traumatic event during the last year^j^				
No	364 (43.2)	175 (42.7)	189 (43.8)	0.808
Yes	478 (56.8)	235 (57.3)	243 (56.3)	
Cognitive status^k^				
Normal	643 (75.4)	340 (80.4)	303 (70.5)	<0.001
Slight deficit	149 (17.5)	68 (16.1)	81 (18.8)	
Cognitive impairment	61 (7.2)	15 (3.5)	46 (10.7)	

In the qualitative variables, *n* (%) is represented in each category. SD: standard deviation; BMI: body mass index (kg/m^2^).

Missing values.

^a^14;

^b^11;

^c^41;

^d^1;

^e^1;

^f^1;

^g^1;

^h^11;

^i^10;

^j^13;

^k^2.

### Sample size estimation

The sample size required for our analysis was calculated to assess the predictive capacity of frailty instruments for adverse events, as part of a subsequent cohort study [10]. This calculation showed that a minimum sample size of 650 subjects (total for the two cohorts) was needed. This minimum requirement was then exceeded by 40% to allow for possible losses to follow-up, resulting in a final requirement of 450 subjects per study group.

### Statistical analysis

The data were entered into a Microsoft Access database (Version 2013) and analysed using SPSS for Windows, Version 16.0. (SPSS Inc., Chicago, IL, USA). The continuous variables are presented with means and standard deviations (SD), and categorical ones by frequencies and percentages (%). Differences between the groups were evaluated using Student’s *t*-test for continuous variables (Mann–Whitney U test for non-normal distributions), and the chi-squared test for categorical ones (Fisher’s exact test for expected frequencies <5).

Logistic regression analyses were performed, in bivariate and multivariate fashion, taking the presence of frailty as the outcome variable, and determining the odds ratios (OR) with 95% confidence intervals (95%CI) of the independent variables. In the multiple logistic regression analysis ‘geographical area’ was used as the first step, and in a second stepwise block (both forward and backward to obtain a more parsimonious model), the independent variables found to be statistically significant in the prior bivariate analysis were introduced, conditioned by selecting the model with the fewest variables and strongest goodness of fit. For the categorical variables, a value of 1 was assigned to the reference category. Goodness of fit was assessed using the Hosmer–Lemeshow test, and the variance of the model was explained by Nagelkerke’s R^2^. In all analyses, the level of statistical significance was set at *P* <0.05.

### Research ethics

The project was approved by the corresponding research ethics committees (CEIC Euskadi 01/2015 and CEI Costa del Sol 11/2014). The data was registered anonymously, in strict accordance with applicable data protection laws and regulations. This study was supported by public grants from Instituto de Salud Carlos III and co-funded by the European Regional Development Fund.

## Results

### Participants

Of the 885 individuals initially included, 20 did not meet the age and Barthel criteria and they were excluded. In another 10 patients, the frailty identification criteria could not be assessed. Thus, the final study sample consisted of 855 subjects, 432 (50.5%) from the Costa del Sol and 423 (49.5%) from Gipuzkoa. The participants’ characteristics are summarized in Table 2.

### Prevalence of frailty and population characteristics

Frailty prevalence in the total sample was 26.2% (95%CI: 23.2–29.2). In Gipuzkoa, the prevalence was 14.2% (95%CI: 10.7–17.6), and in Costa del Sol it was 38% (95%CI: 33.3–42.7). The participants’ sociodemographic characteristics, lifestyle habits and health status are summarized in Table 2, both for the total population and for each study area.

### Frailty-related factors

The results for the frailty-related factors, according to bivariate and multiple logistic regression, are shown in Table 3. In addition to the above differences between the study areas (raw OR: 3.7, 95%CI: 2.65–5.18), differences were also observed between the sexes, with women being more likely to present frailty (OR: 2.08, 95%CI: 1.51–2.86).

**Table 3.** Simple and logistic regression according to the presence of frailty.

	Bivariate				Multivariate			
Total	OR	95%CI		*P*	OR	95%CI		*P*
		Lower	Upper			Lower	Upper	
Area								
Gipuzkoa	1.00			<0.001	1.00			<0.001
Costa del Sol	3.70	2.65	5.18		3.51	2.29	5.36	
Sex								
Male	1.00			<0.001	1.00			<0.001
Female	2.08	1.51	2.86		1.98	1.37	2.86	
Age								
≤80	1.00			0.548	–			
>80	1.11	0.80	1.53					
Education background								
None or only primary	0.95	0.65	1.40	0.795	–			
Secondary or university	1.00							
Marital status								
Married—civil partnership	1.00			0.230	–			
Single	1.10	0.52	2.32					
Separated—divorced—widowed	1.33	0.96	1.84					
Family income								
≤€1200	1.36	0.98	1.89	0.063	–			
>€1200	1.00							
BMI								
	1.06	1.02	1.09	0.002	–			
Smoking								
Non-smoker	1.86	0.91	3.80	0.024				
Ex-smoker	1.25	0.60	2.60		–			
Smoker	1.00							
Cumulative illness rating scale (0–56)								
	1.17	1.12	1.23	<0.001	1.05	1.00	1.10	0.04
Number of medicinal drugs taken								
	1.17	1.11	1.22	<0.001	–			
Self-perceived life style								
Healthy	1.00			<0.001	1.00			0.012
‘Intermediate’—unhealthy	4.58	3.05	6.86		3.37	2.05	5.54	
Health status (0–100)								
	*0.97*	*0.96*	*0.98*	<0.001	0.96	0.95	0.97	<0.001
Satisfaction with the domestic environment								
Satisfied	1.00			<0.001	1.00			0.012
Unsatisfied	3.26	2.02	5.25		2.11	1.18	3.76	
Traumatic event during the last year								
No	1.00			0.116	–			
Yes	1.29	0.94	1.76					
Cognitive status								
Normal	1.00			<0.001	1.00			<0.001
Slight deficit	2.24	1.53	3.29		2.07	1.31	3.26	
Cognitive impairment	5.52	3.20	9.52		4.10	2.05	8.19	

BMI: body mass index (kg/m^2^). In the quantitative variables, the increase in risk is expressed per unit of corresponding scale.

Multivariate model fit (*n* = 801), Hosmer–Lemeshow (*P* = .665), Nagelkerke’s *R*^2^ = 0.519.

The multivariate model was obtained by step-forward methods with a sample of 801 individuals (the variables included in the model were shown to be identical by replicating the analysis using the step-backward method). The goodness of fit was acceptable (*P* = 0.665) and the level of explained variance was 0.519. The geographical area was included in the model, with an adjusted OR of 3.51 (95%CI: 2.29–5.36) for the Costa del Sol area to Gipuzkoa. Sex differences were also included; thus, women were at greater risk of frailty, with an adjusted OR of 1.98 (95%CI: 1.37–2.86). Increased frailty prevalence was also related to the CIRS index of comorbidity (OR: 1.05; 95%CI: 1.00–1.10; which means an increase in risk of 5% for each unit on the scale), whereas self-perceived functional health status was a protective factor (OR: 0.96; 95%CI: 0.95–0.97; in terms of risk, an increase of 4% for each point less on the scale). The presence of frailty was higher among those who perceived their health to be poor (OR: 3.37; 95%CI: 2.05–5.54), those who were dissatisfied with their domestic environment (OR: 2.11; 95%CI: 1.18–3.76) and those who had an inadequate cognitive status, regarding both full cognitive impairment (OR: 4.1; 95%CI: 2.05–8.19) and slight cognitive deficit (OR: 2.07; 95%CI: 1.18–3.76).

## Discussion

### Main findings

The analyses revealed a prevalence of frailty (26.2%) in this sample of individuals aged 70 years and over living independently in the community. Frailty presence was higher among women, those who perceived their lifestyles to be unhealthy, those unsatisfied with their domestic environment, and those with an impaired cognitive status. The prevalence of frailty was three times as high among participants living in the southern PHC area (Costa del Sol) than in the northern one (Gipuzkoa).

### Limitations

A limitation of the present study is that the weakness criterion of the Fried phenotype was measured by the timed get up and go test, and not with a dynamometer adjusting the values according to BMI and sex. However, the Fried criteria are frequently implemented with modified items [14,15].

### Results in relation to existing literature

*Geographical differences.* The prevalence of frailty (26.2%) in this population of people over 70 is practically the median of the prevalence range identified in a recent systematic review (4–59.1) [4]. Focusing on comparisons with each PHC area, studies, which have evaluated frailty using Fried’s modified phenotype, have found prevalence rates similar to those in Gipuzkoa (14%). Thus, the FRALLE survey reported a prevalence of 10% in Lleida (North East Spain) [16]; another study reported 11% in urban areas to the north of Madrid [14], and the FRADEA study reported 17% in Albacete [15]. Similar studies have been carried out in developed regions in other European countries, for example, the Bordeaux three-city study (reported prevalence of frailty: 18%) conducted in southern France [17]. In the Costa del Sol area, the prevalence of frailty according to the modified Fried phenotype (38%) is higher than that reported in previous studies conducted in developed European countries using a comparable methodology; however, similar results have been reported in Latin America, such as the 39% obtained in a study carried out in a Mexican population living in urban areas [18], and the value of 37% reported in the SABE macro study carried out in cities in Latin America and the Caribbean [19].

At an ecological level, the differences between the two study areas may be due to our evaluation of areas presenting different socioeconomic levels. As Campbell and Buchner pointed out, frailty is a multi-systemic syndrome in which the interaction between individuals and their environment is a key factor [20]. Thus, a possible explanation in ecological terms is that Gipuzkoa forms part of the Basque Country in northern Spain, which is one of the most highly developed areas in the EU, by GDP per capita, whilst the Costa del Sol in Andalusia (southern Spain), is classed as a transition region; the resulting differences in social and health spending may affect levels of frailty within the population. An ecological study in several European countries recorded a strong correlation between a country’s economic indicators and its level of frailty [21].

*Individual differences.* Regarding the individual, there were gender-specific differences, with women being twice as likely as men to present frailty; this finding is consistent with previous studies [4,6].

Our finding of an inverse relationship between frailty and perceived quality of life is in line with a previous meta-analysis [22], which included studies with a comparable design and level of statistical power [23,24]. Also relevant to our multivariate analysis is the positive relationship reported in previous research between frailty and cognitive impairment and between frailty and multimorbidity [25,26].

Although smoking is not significantly associated with frailty, smokers and ex-smokers tend to present less frailty than non-smokers (20% and 30%, respectively), values that are similar to those reported in a prevalence study of similar design conducted in Mexico [27]. This pattern might be explained by the presence of Neyman bias [28], which affects research designs in which prevalent cases are evaluated.

Although age is the most frequently correlated sociodemographic characteristic, in a positive sense, with frailty [29], this relationship was not detected in our 70+ sample.

### Implications

The considerable difference observed in the presence of frailty between the two study areas, according to individual-related variables, highlights the existence of geographical inequalities; therefore, action should be taken by public health authorities to promote screening and healthcare interventions.

## Conclusion

A high prevalence of frailty was observed in a sample of men and women aged 70 years and over, and living independently in the community. This prevalence was three times as high in a region with a relatively low economic level. Individual factors correlated with frailty were female sex, comorbidity, poorer self-perceived lifestyle and health status, and dissatisfaction with the domestic environment.

## Supplementary Material

Supplementary Material: Barthel Index Activity
