# Supplementary material for: Prevalence of frailty among community-dwelling elderly persons in Spain and factors associated with it
Source: Eur J Gen Pract. 2019 Oct 22;25(4):190–6. doi: 10.1080/13814788.2019.1635113 (PMC6853242; doi:10.1080/13814788.2019.1635113)
Supplement: Supplementary Material: Barthel Index Activity [file IGEN_A_1635113_SM6164.docx]

**Appendix A**

**Barthel Index Activity (original in Spanish)**

| FEEDING  0 = unable  5 = needs help cutting, spreading butter, etc., or requires modified diet  10 = independent |
| --- |
| BATHING  0 = dependent  5 = independent (or in shower) |
| GROOMING  0 = needs to help with personal care  5 = independent face/ hair/ teeth/ shaving (implements provided) |
| DRESSING  0 = dependent  5 = needs help but can do about half unaided  10 = independent (including buttons, zips, laces, etc.) |
| BOWELS  0 = incontinent (or needs to be given enemas)  5 = occasional accident)  10 = continent |
| BLADDER  0 = incontinent, or catheterized and unable to manage alone  5 = occasional accident  10 = continent |
| TOILET USE  0 = dependent  5 = needs some help, but can do something alone  10 = independent (on and off, dressing, wiping) |
| TRANSFERS (BED TO CHAIR AND BACK)  0 = unable, no sitting balance  5 = major help (one or two people, physical), can sit  10 = minor help (verbal or physical)  15 = independent |
| MOBILITY (ON LIVEL SURFACES)  0 immobile or < 50 yards  5 = wheelchair independent, including corners, > 50 yards  10 = walks with help of one person (verbal or physical) > 50 yards  15 = independent (but may use any aid; for example, stick) > 50 yards |
| STAIRS  0 = unable  5 = needs help (verbal, physical, carrying aid)  10 = independent |
